# Mitigation of radiation-induced jejunum injuries in rats through modulation of the p53-miR34a axis using etoricoxib-loaded nanostructured lipid carriers

**DOI:** 10.1038/s41598-024-73469-7

**Published:** 2024-10-10

**Authors:** Noha Sayed Hamed, Sahar Khateeb, Shady A. Elfouly, Amina M. A. Tolba, Amal I. Hassan

**Affiliations:** 1 Radioisotopes Department, Nuclear Research Centre, Egyptian Atomic Energy Authority (EAEA), Cairo, 11787 Egypt; 2 Biochemistry Division, Department of Chemistry, Faculty of Science, Fayoum University, Fayoum, Egypt; 3 Anatomy Department, Faculty of Medicine, Al-Azhar University, Girl’s Branch, Cairo, Egypt

**Keywords:** Etoricoxib-nanostructured lipid carriers, Oxidative stress, Inflammation, Apoptosis, Radioprotection, Gastrointestinal toxicity, Biochemistry, Biotechnology, Drug discovery, Molecular biology

## Abstract

The most widely used cancer therapy is radiation therapy, but radiation damage to healthy tissues, particularly the gastrointestinal (GI) system, frequently reduces its effectiveness. This study investigates whether etoricoxib-loaded nanostructured lipid carriers (Et-NLC) could help shield the rat jejunum from radiation damage. Gamma irradiation (6 Gy) was used to damage the jejunum of Wistar albino rats, and then Et or Et-NLC (10 mg/kg b.w.) was administered orally for 14 days. It was found that the amounts of glutathione S-transferase (GST), superoxide dismutase (SOD), and nitric oxide (NO) decreased after irradiation but increased after Et-NLC therapy. Molecular analysis showed radiation-induced expression of microRNA-34a (miR34a), which may be involved in cellular stress response. Et-NLC treatments modulated the expression of miR34a, suggesting possible regulatory roles. Western blot analysis revealed changes in P53, interleukin-6 (IL-6), interleukin-10 (IL-10), tumor necrosis factor-alpha (TNF-α), and cyclooxygenase-2 (COX-2) levels. Et-NLC treatments decreased TNF-α, IL-6, IL-10, and COX-2 levels, indicating anti-inflammatory actions. DNA fragmentation analysis revealed a decrease in apoptotic activity after Et-NLC treatments. A histopathological examination confirmed that Et-NLC treatments had attenuated radiation damage, which had improved vascularization and reduced inflammation. The findings show that Et-NLC is more effective than Et-alone at reducing damage to the jejunum caused by radiation by controlling inflammation, oxidative stress, and apoptotic activity.

**Supplementary Information:**

The online version contains supplementary material available at 10.1038/s41598-024-73469-7.

## Introduction

Radiotherapy is a common choice for cancer treatment, but it often causes toxicities in normal tissues, limiting its effectiveness against tumors. Radiation may damage several organs, including bone marrow, kidneys, the gut, and lungs^1,2^. The intestine is particularly susceptible to radiation-induced damage, which hinders the use of radiation therapy for abdominal and pelvic tumors^3,4^. This intestinal toxicity, known as radiation enteropathy, can manifest as early, acute, or delayed/chronic symptoms following radiotherapy^3,5^.

Previous investigations have demonstrated that ionizing radiation (IR) induces cell oxidative damage, resulting in tissue and organ dysfunction^6^. Inflammatory responses and the chronic production of reactive oxygen species (ROS) are implicated in radiation-induced intestinal damage. Small intestinal cell irradiation can lead to sustained oxidative damage due to mitochondrial dysregulation and upregulation of NADPH oxidase^7^. Increased expression of inflammatory mediators such as cyclooxygenase-2 (COX-2) worsens the damage to the intestines after radiation exposure^8^. High levels of cytokines that cause inflammation, such as IL-1, IL-6, IL-8, and TNF-α are present when IR damages the gut^9^. Reducing chronic inflammation and oxidative stress activation is suggested to lessen radiation damage to organs such as the small intestine^10^.

Radiation-induced intestinal damage negatively impacts patient treatment outcomes and quality of life, underscoring the importance of identifying more effective compounds for intestinal radiation injury treatment.

Various pharmacological categories, such as growth factors, protease inhibitors, angiotensin-converting enzyme (ACE) inhibitors, isoflavones, COX-2 inhibitors, and nonsteroidal anti-inflammatory medicines (NSAIDs), have been studied as prospective substances that might reduce the harmful effects of radiation^11^. Arcoxia is the commercial name for etoricoxib (Et). It is now permitted in more than 62 nations worldwide, but not in the United States^12–14^. Et is a widely used COX-2 selective inhibitor, and previous studies have shown its effectiveness in alleviating inflammation in patients with rheumatoid arthritis and other systemic inflammatory diseases^15^. Additionally, Huang and Tso^16^ reported that Et is a selective cyclooxygenase-2 inhibitor with a lower risk of gastrointestinal toxicity compared to traditional NSAIDs. Et, a NSAID used for pain relief and inflammation management, is often prescribed for cancer treatment with radiotherapy^17–19^. However, its oral intake can be adverse and has poor solubility^20,21^. The GI toxicity of selective COX-2 inhibitors may depend on the dose and duration of treatment^22^. Despite the potential of some NSAIDs like etoricoxib (Et) in mitigating radiation-induced gastrointestinal toxicity, there is still a need to explore alternative compounds and formulations that could offer improved efficacy, reduced side effects, or novel mechanisms of action^23^.

Nanostructured lipid carriers (NLC) are a promising way to deliver drugs because they are biocompatible and biodegradable on a physical and chemical level. NLC, composed of solid and liquid lipids, is designed to create a less ordered lipidic core, influencing the final product’s properties^24^. NLCs have advantages over polymeric methods for drug delivery since their lipid structure is similar to the lipid structure of our biomembranes. Lipids’ biocompatibility and biodegradability make them ideal carriers for intravenous delivery, but other synthetic and semi-synthetic polymeric carriers are not. Since NLCs are non-toxic even after systemic administration, they may be used for diagnostic purposes. These carriers have a highly adaptable surface, allowing for pinpoint accuracy in aiming^25^. NLCs were selected for this study because of their exceptional pharmaceutical and drug delivery characteristics, which include improved oral bioavailability of poorly water-soluble drugs^26^. The p53-miR34a axis is known to play a crucial role in the cellular response to radiation-induced stress and DNA damage. Activation of p53 leads to the upregulation of miR34a, which is a well-characterized target of p53 and a key regulator of apoptosis, cell cycle arrest, and senescence^27,28^. Increased expression of miR34a has been associated with radiation-induced injury in various tissues, including the intestine^29^. This suggests that modulation of the p53-miR34a axis may be an essential mechanism by which Et-NLC could exert its protective effects against radiation-induced jejunal damage. Further investigation of the changes in miR34a expression and its upstream regulation by p53 in response to radiation and Et-NLC treatment could provide valuable insights into the underlying molecular mechanisms.

This study examines how the nanostructured lipid carrier (NLC) formulation of etoricoxib (Et-NLC) can improve its oral bioavailability, and efficacy, and mitigate gastrointestinal toxicity compared to the conventional Et formulation. The focus is on evaluating the ability of Et-NLC to reduce radiation-induced inflammation, oxidative stress, and apoptosis in the jejunum, while modulating the p53-miR34a axis, thereby minimizing jejunum toxicity relative to traditional Et treatment.

## Results

### Blood serum biomarkers

Compared to the control group, the γ-radiation group (R) had much higher levels of urea (71.00 ± 8.39) by 88.53% and creatinine (1.000 ± 0.05) by 274.53% (*p* < 0.05) (Table 1). Additionally, Et treatment induced a noteworthy elevation in urea (63.00 ± 6.80) and creatinine (0.50 ± 0.01) compared to the control group (*p* < 0.05). Conversely, treatment with Et-NLC following irradiation resulted in a reduction in the levels of urea (51.00 ± 3.51) and creatinine (0.566 ± 0.03) (*p* < 0.05) (Table 1).

**Table 1:** Urea, creatinine, AST, and ALT levels.

Treatments	Creatinine	Urea	AST	ALT
Control	0.267 ± 0.03^c^	37.66 ± 1.45^b^	49.33 ± 8.08^c^	27.66 ± 2.03^c^
Et	0.500 ± 0.01^b^	63.00 ± 6.80^a^	50.33 ± 10.17^c^	24.33 ± 1.48^c^
Et-NLC	0.633 ± 0.03^b^	46.67 ± 2.23^b^	52.66 ± 4.33^c^	22.00 ± 1.21^c^
R	1.000 ± 0.05^a^	71.00 ± 8.39^a^	196.0 ± 2.64^a^	138.66 ± 2.03^a^
Et-R	0.633 ± 0.030^b^	58.33 ± 2.19^ab^	96.66 ± 7.26^b^	78.33 ± 3.75^b^
Et-NLC*-*R	0.566 ± 0.030^b^	51.00 ± 3.51^ab^	52.66 ± 4.33^c^	22.00 ± 2.17^c^
F-Value	43.9	5.37	76.61	247.40
P	0.001	0.008	0.001	0.001

Results are the mean ± SE (*n* = 6). Values in the same column with the different superscript are significantly at *P* < 0.05, one-way ANOVA then Tukey’s post-hoc comparison.

Higher levels of AST and ALT were found in the radiation-exposed group compared to the control group, by 196.00 ± 2.64 by 146.67% and 138.66 ± 2.03 by 401.30% (*p* < 0.05), respectively (Table 1). AST and ALT levels caused by radiation got better when Et-R was given. They went down to 96.66 ± 7.26 (50.68%) and 78.33 ± 3.75 (43.51%), respectively. The treatment with Et-NLC-R also showed similar improvements, lowering these enzyme levels to 52.66 ± 4.33 (73.13%) and 22.00 ± 2.17 (84.13%), respectively, compared to the irradiation group (Table 1).

### Biomarkers in the jejunum homogenate

#### NO and MDA levels

**Fig. 1:**
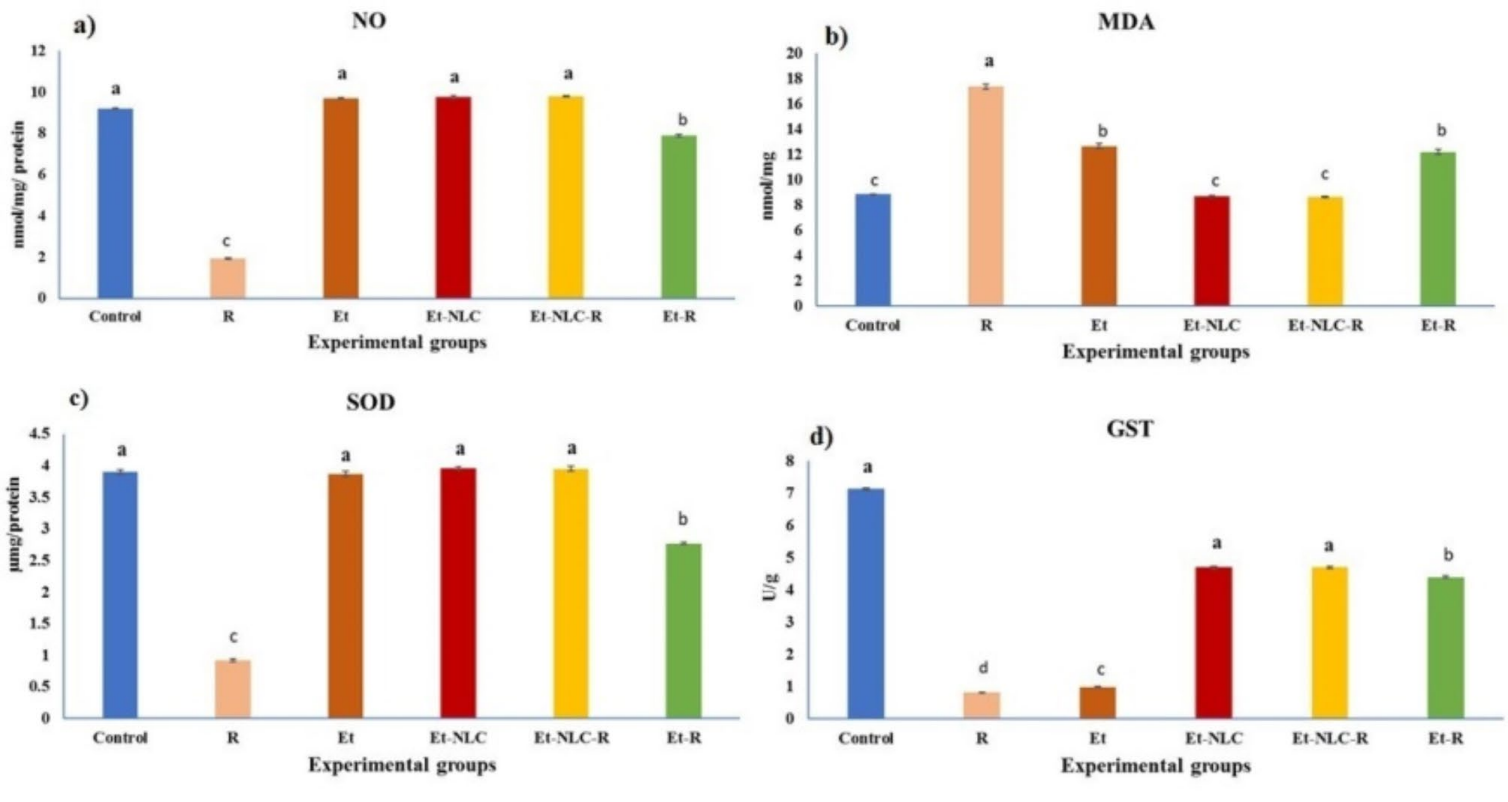
Effects of Et-NLC treatment on, (**a**) NO, (**b**) MDA, (**c**) SOD, and (**d**) GST in jejunum homogenate. The results are the mean ± SE (*n* = 6). Values with the different superscript are significantly at *P* < 0.05 (one-way ANOVA then Tukey’s post hoc comparison).

The γ-irradiation significantly decreased the concentrations of NO (9.20 ± 0.09) to (1.92 ± 0.02) by 79.02% and increased of MDA (8.58 ± 0.11) to (17.35 ± 0.91) by 96.05% (*p* < 0.05) compared to controls (Fig. 1a and b). Treatment with Et-NLC-R showed a significant decrease for MDA (8.65 ± 0.31) by 50.14% (*p* < 0.05) for MDA levels. The data demonstrated that the treatment with Et-NLC-R significantly elevated the levels of NO to 407.77 in comparison to the group that was exposed to radiation and left without treatments.

#### SOD, GST, COX-2, and P53 levels

As shown in Fig. 1c and d, the levels of SOD and GST were significantly decreased in the radiation-exposed group compared to the control group, decreasing by 76.41% and 88.50%, respectively (*p* < 0.05). When Et-R was given, the levels of SOD and GST that were caused by radiation went up, with changes of up to 201.10% and 436.56%, respectively. Notably, the treatment with Et-NLC-R exhibited even more substantial improvements in these antioxidants, showing percentage changes of 329.35% for SOD and 473.17% for GST compared to the irradiation group. These results indicate that Et-NLC may have a more substantial protective effect in reducing the adverse impact of radiation-induced oxidative stress.

**Fig. 2:**
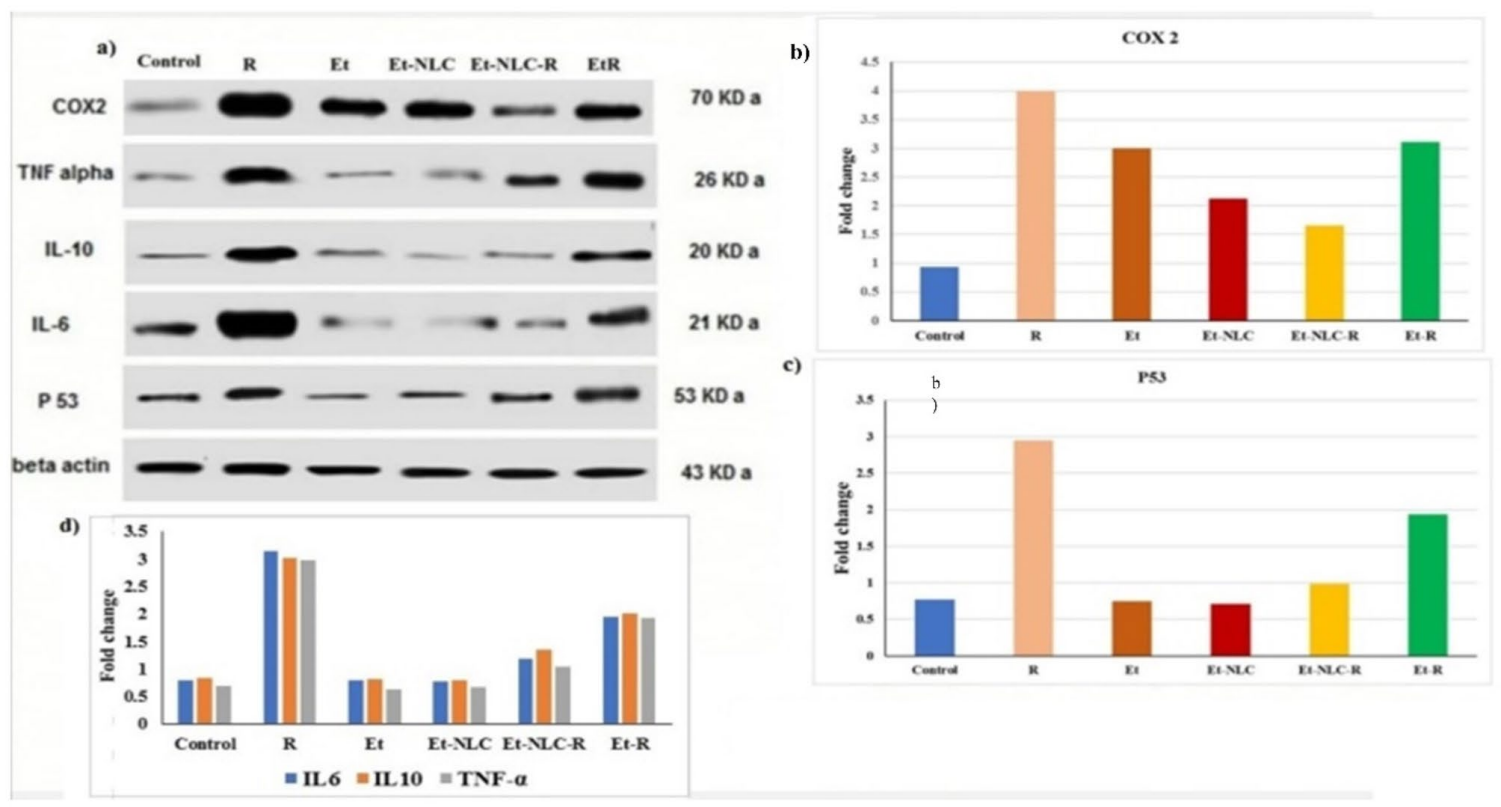
Western blot analysis shows (**a**) the expression of COX-2, P53, and cytokines (IL-6, IL-10, and TNF-α) in jejunum homogenate, (**b**) COX2, (**c**) P53, and (**d**) cytokines (TNF-α, IL-6, and IL-10).

The Western blot test (Fig. 2a) shows that the jejunum homogenate has COX-2, P53, and cytokines (IL-6, IL-10, and TNF-α) that are expressed. The effect of nanostructured lipid carrier-loaded Et (Et-NLC) and radiation (R) on COX-2 and P53 expression in a rat model of radiation-induced jejunum injury is shown in Fig. 2b and c. Radiation increased COX-2 expression by 4.0 fold (330.11%) and increased P53 expression by 2.95 fold (283.12%) (*p* < 0.05) compared to normal controls. However, the combination treatment of Et-NLC and radiation (Et-NLC-R) led to a significant decrease in COX-2 to 1.66 fold (58.50%) and a decrease in P53 0.99 fold (66.44%), respectively, compared to radiation alone.

#### IL6, IL10, and TNF-α levels

Figure 2d displays how radiation (R), Et-R, and Et-NLC-R affect the inflammatory cytokines IL-6, IL-10, and TNF-α. Compared to control, radiation treatment significantly increased IL-6 by 3.15 fold (288.90%), IL-10 by 3.02 fold (255.30%), and TNF-α by 2.99 fold (327.14%) (*p* < 0.05). However, the combination of nanoformulated drug and radiation (Et-NLC-R) markedly reduced radiation-induced cytokine increases. Et-NLC-R decreased IL-6 by 1.2 fold (61.90%), IL-10 by 1.36 fold (54.97%), and TNF-α by 1.05 fold (64.88%) compared to control radiation (*p* < 0.05).

### DNA fragmentation and miR34a expression profiles

**Fig. 3:**
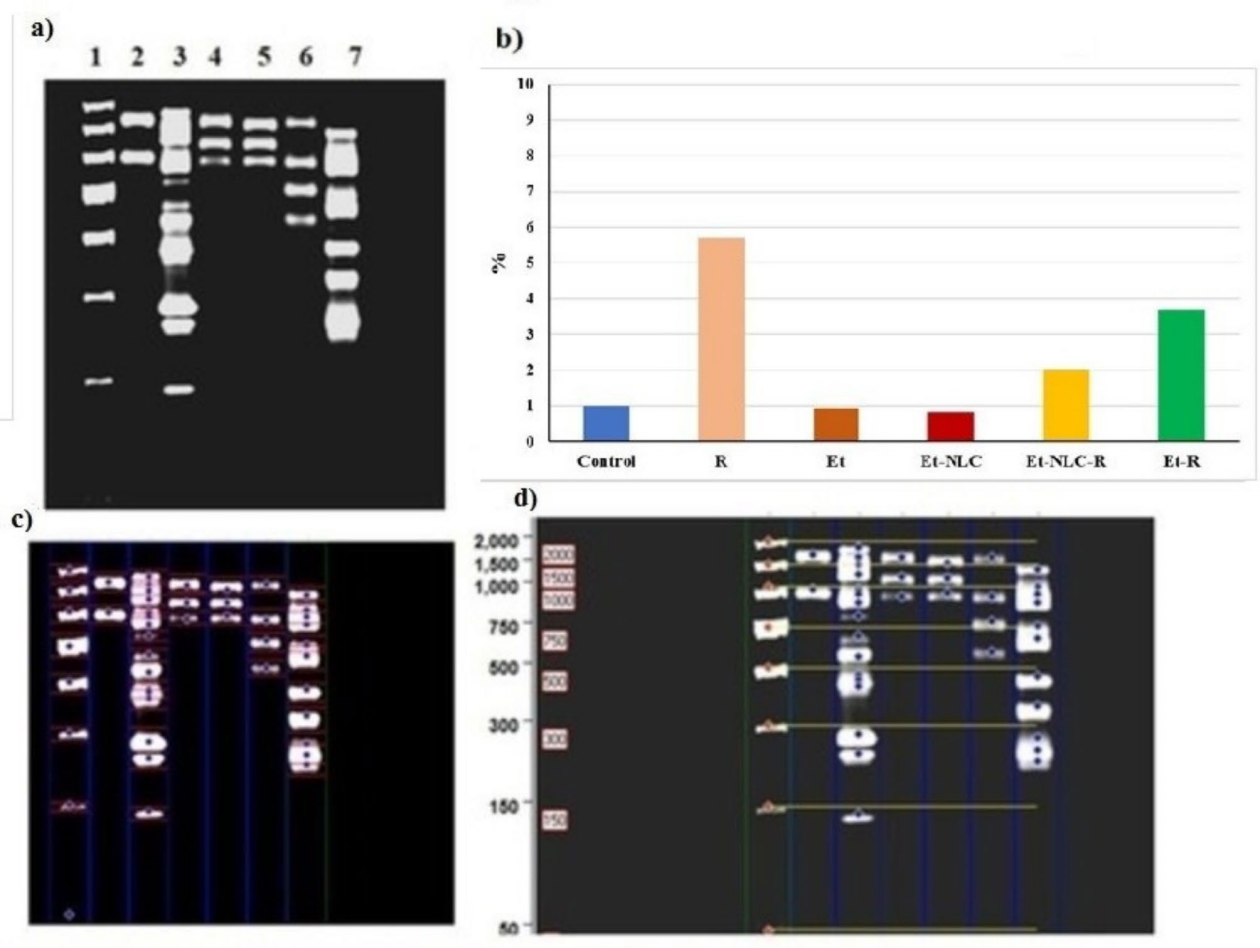
(**a**) DNA fragmentation patterns for intestine samples, lane 1: DNA ladder, lane 2: control, lane 3: R, lane 4: Et, lane 5: Et-NLC, lane 6: Et-NLC-R, lane 7: Et-R, b) Fragments number for patterns for intestine samples, (**c,d**) Computerized detection for fragmentation patterns for intestine samples.

DNA fragmentation analysis revealed a decrease in apoptotic activity in the Et-NLC treatment group compared to the radiation-exposed group (Fig. 3a–d). This suggests that the Et-NLC treatment was able to attenuate the radiation-induced apoptosis in the rat jejunum.

**Fig. 4:**
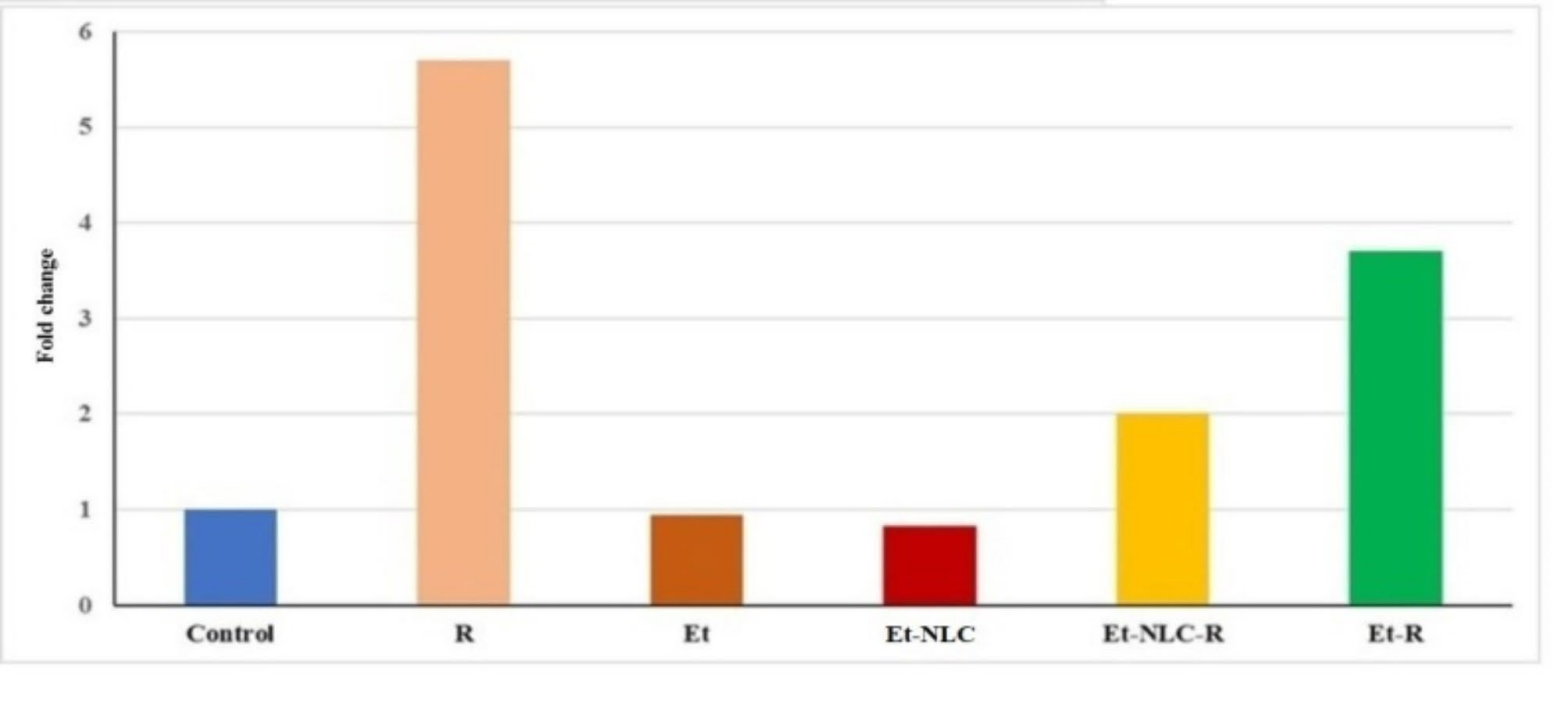
Effects of Et-NLC treatment on miR34a relative quantity expression.

Molecular analysis showed that gamma irradiation induced the expression of miR34a (Fig. 4). miR34a has been implicated in cellular stress response pathways. Interestingly, the Et-NLC treatments were able to modulate the expression of miR34a compared to the radiation-exposed group, indicating a potential regulatory role of the nanocarrier formulation on this key microRNA.

miR34a a pivotal regulator of cellular processes including apoptosis, proliferation, and stress response, was assessed to unravel the impact of treatments on its expression levels. The baseline expression level miR34a in the control group was 0.998 (Fig. 4). Exposure to γ-irradiation alone led to a substantial increase in miR34a expression, with an average fold change of 5.7 compared to the control group. Treatment with Et in the absence of radiation led to a mean fold change of 0.95 in miR34a expression compared to the control group. Administration of Et-NLC reduced miR34a expression level, with a mean fold change of 0.83 compared to the control group. Post-radiation treatment with Et-NLC exhibited an altered miR34a expression profile, with a mean fold change of 2.01 compared to the control group. Similarly, treatment with Et following radiation exposure led to a substantial elevation in miR34a expression, with a mean fold change of 3.71 compared to the control group. These mean fold changes highlight the relative alterations in miR34a expression in response to radiation and its modulation by Et-based treatments. These findings provide insights into the regulatory effects of these treatments on miR34a and its potential role in the cellular response to radiation-induced stress.

### Histopathological findings

**Fig. 5:**
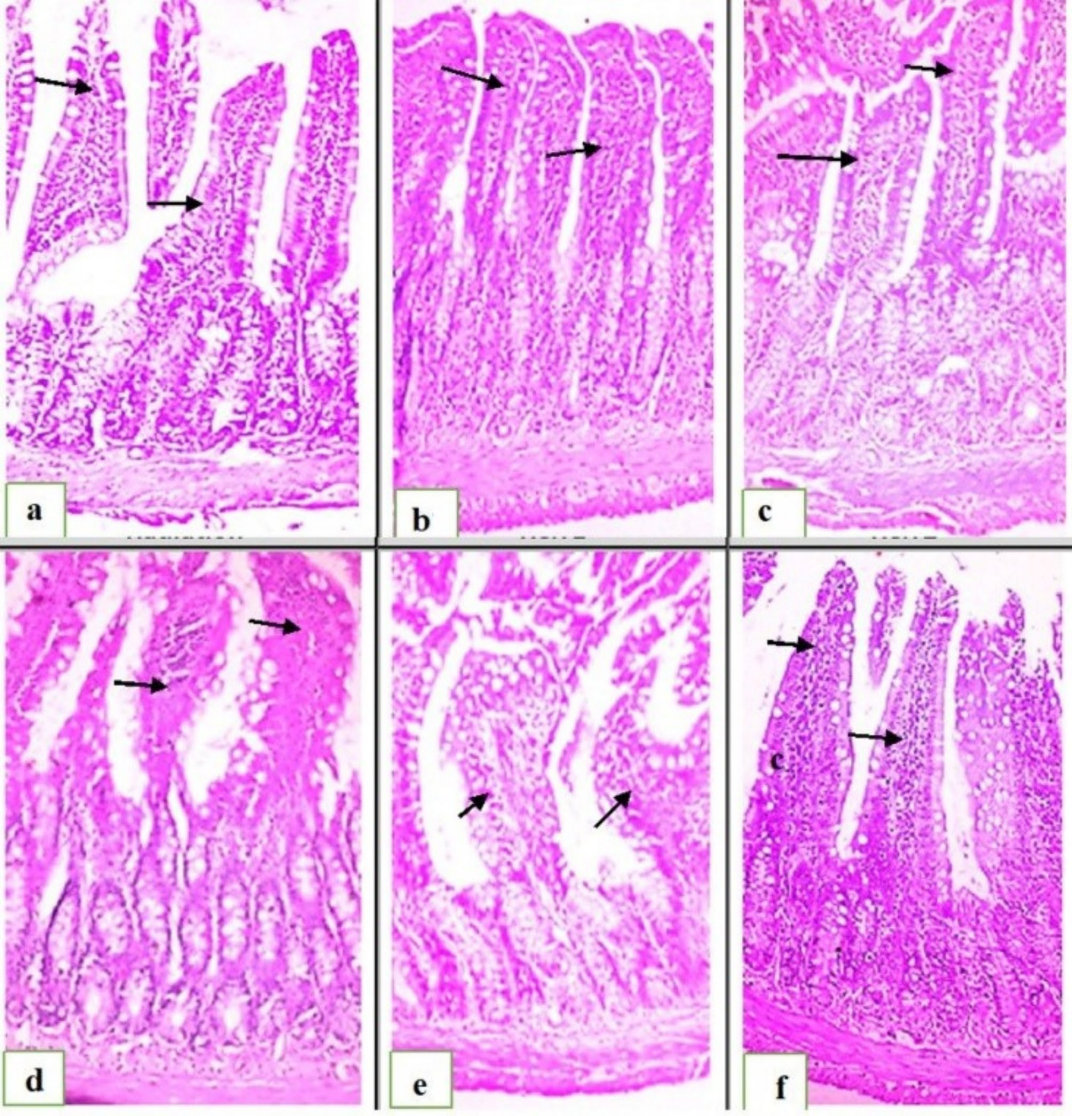
Photomicrograph of a cross-section in the jejunum showing (**a**) Control rat, (**b**) Et, (**c**) Et-NLC, (**a–c**) Normal histological structure of intestinal mucosa and villi arrow (H&E ×100) (**d**) Effect of radiation on the intestine of rats (R), massive necrosis of intestinal villi and loss of goblet cells arrow (H&E ×100) (**e**) Effect of Et on irradiated rats (Et-R), epithelial shedding at the tip of some intestinal villi arrow, (**f**) Effect of Et-NLC on irradiated rats (Et-NLC-R), intact villus epithelial lining arrow (H&E ×100).

A light microscope was used to look at sections of the jejunum from the control, Et, and Et-NLC groups. These sections showed normal histological features of the intestinal mucosa. Standing on top of one another, the tall columnar epithelial cells had oval basal nuclei, giving the appearance of finger-like extensions that were the villi. The columnar cells had a score of zero, and several goblet cells dispersed among them. Columnar epithelial cells lined the crypts of the intestines, and their arrangement was regular (score 0). There were no noticeable pathological changes to the intestinal glands, which seemed uninjured and bordered with high cuboidal epithelial cells. (score 0) (Fig. 5a–c).

In the animals exposed to radiation, the intestinal mucosa exhibited severe damage to the villi, characterized by necrosis and hyperactivity or loss of goblet cells (score 3). Intestinal crypts showed shedding of their epithelial lining (score 3). The mucosa and submucosa were infiltrated with numerous mononuclear cells, primarily lymphocytes and macrophages (score 2). Intestinal glands showed necrosis of their epithelial lining (Fig. 5d); however, the intestinal mucosa improved in the animals exposed to radiation and treated with Et. A few epithelial shedding cells were present at the tips of some intestinal villi (score 1), with a standard lining of intestinal crypts (score 0). A few mononuclear cells infiltrated the mucosa and submucosa (score 1). Intestinal glands had an intact epithelial lining (Fig. 5e).

On the other hand, the group of animals exposed to radiation and treated with Et-NLC showed marked improvement compared to the group treated with Et. Numerous goblet cells were scattered along the intestinal villi (score 0), with a standard lining of intestinal crypts (score 0). The submucosa and the mucosa showed slight inflammatory cell infiltration (scoring 1). The intestinal glands had basal basophilic nuclei and an intact epithelial lining (Fig. 5f).

## Discussion

Radiation therapy is a widely used technique in the treatment of cancer, but its efficacy is often limited by adverse effects on normal tissue^30^. Radiation can have significant effects on the gastrointestinal (GI) tract, particularly the jejunum, in cancer patients receiving radiotherapy. These effects can lead to a reduction in quality of life^3^. This study examined the potential mitigating effects of Et-NLC as an alternative treatment modality regarding radiation-induced jejunum injury in rats.

The histopathological examination of jejunum segments provided valuable insights into the protective effects of Et and Et-NLC treatments. When rats were exposed to radiation without any treatment, the villi were severely damaged, the lining of the intestinal crypts shed, and inflammatory cells migrated in. These findings are consistent with previous studies highlighting radiation’s damaging effects on intestinal mucosa^31^.

However, rats that were exposed to radiation and then given Et had better intestinal mucosa, fewer epithelial shedding cells, a normal lining of the intestinal crypts, and less inflammation. Notably, the group that was treated with Et-NLC did much better than the group that was treated with Et. There were more goblet cells along the villi, the intestinal crypts were still intact, and there were fewer inflammatory cells in the gut. These results suggest that Et-NLC has a more pronounced protective effect against radiation-induced intestinal damage than Et-alone.

Liver function, as assessed by measuring blood ALT and AST levels, constitutes a significant determinant of the radiation-induced injury. The findings of our study revealed a notable increase in ALT and AST levels in untreated irradiation mice, suggesting the presence of liver injury. This finding is consistent with previous studies reporting hepatic injury following radiation exposure^32^. Etoricoxib has been shown to effectively inhibit oxidative stress and inflammatory markers in the kidney after ischemia-reperfusion insult^33^. Similarly, recent studies have shown that etoricoxib can significantly inhibit elevated levels of COX-2, malondialdehyde (MDA), myeloperoxidase (MPO), and induce anti-inflammatory enzymes such as GSH have demonstrated, both in ischemia and reperfusion conditions, in etoricoxib liver it has been found to alter the oxidant/antioxidant balance, thereby improving the overall antioxidant status, in the context of ischemia reperfusion injury^34^. Collectively, these data this suggests that the extension of the cytoprotective properties of etoricoxib beyond the conduit and relevant reperfusion of ischemia in various organs may be a valuable strategy for reducing tissue damage. Recent study demonstrated that Et in mitigating acute kidney injury and intestinal damage induced by ionizing gamma radiation^35^. Our findings on the protective effects of Et against radiation-induced jejunum injury align with previous studies on etoricoxib’s anti-inflammatory properties. For instance, Huang and Tso^16^. reported that etoricoxib improved osteoarthritis pain relief and joint function, indicating its effectiveness in reducing inflammation. Similarly, our results showed that Et treatment led to improvements in AST and ALT levels induced by radiation, suggesting a hepatoprotective effect.

However, treatment with Et-NLC post-radiation exposure significantly reduced ALT levels compared to the irradiated group, suggesting a protective effect on liver function. This result suggests a potential hepatoprotective impact of Et-NLC even without radiation exposure. The exact mechanism underlying this effect requires further investigation. Estimating serum urea and creatinine levels provides valuable information about kidney function, as these markers are commonly used to evaluate renal toxicity^36^. Our results showed a significant increase in both creatinine and urea levels in rats exposed to radiation without treatment. This elevation indicates renal dysfunction, which is a known side effect of radiation therapy^37^. Importantly, treatment with Et-NLC post-radiation exposure significantly reduced creatinine and urea levels compared to the irradiated group.

The reduction in serum creatinine and urea levels in the Et-NLC treatment groups suggests a protective effect on renal function. Moreover, the enhanced protective effectiveness reported in the Et-NLC treatment group can be attributed to the improved bioavailability and drug distribution^24^.

Examining nitric oxide (NO) and malondialdehyde (MDA) levels can provide insight into oxidative stress and inflammation. Our results showed that treatment with Et-NLC after irradiation significantly increased NO levels and decreased MDA levels compared to the irradiated group. This discovery implies a reduction in inflammation and oxidative stress; two factors that are recognized to amplify radiation-induced tissue damage^38^.

Enzymes like glutathione-S-transferase (GST) and superoxide dismutase (SOD) help prevent oxidative damage to cells. Our results showed that groups that were treated with Et-NLC had much higher levels of SOD after radiation, which suggests that their antioxidant defenses were strengthened. These results point to the antioxidant benefits that COX-2 inhibitors can have^39^. Conversely, GST levels decreased significantly in the Et-NLC treatment groups after radiation exposure compared to the control group. However, there was a significant increase in GST levels in the treated groups compared to the irradiated group. These findings show that different antioxidant systems and Et-NLC’s possible ability to change GST activity are connected in a complex way. The nanostructured lipid carrier-loaded drug plus radiation combination changed COX-2 and P53 expression in rats’ small intestines better than radiation, unformulated drugs, or radiation plus unformulated drugs.

The inflammatory response plays an essential role in radiation injury. Interleukin-6 (IL-6), interleukin-10 (IL-10), tumor necrosis factor-alpha (TNF-α), cyclooxygenase-2 (COX-2), and p53 are molecules that play a big part in the damage and inflammation that radiation does to cells^40^. It is worth noting that the best improvement in IL-6, IL-10, TNF-α, and P53 levels was observed with Et-NLC treatment after radiation exposure. Cytokine profiles like IL-6, IL-10, and TNF-α improved, suggesting that Et-NLC might help the immune system deal with damage to the jejunum caused by radiation. It is a medication that explicitly inhibits the COX-2 enzyme involved in inflammation. It also has qualities that reduce inflammation and counteract the effects of harmful molecules in the body. The observed reduction in inflammatory markers like TNF-α, IL-6, and IL-10 in our Et-treated groups is consistent with the known COX-2 inhibitory mechanism of etoricoxib. This aligns with findings by Laube et al.^41^. who demonstrated etoricoxib’s effectiveness in reducing inflammation in rheumatoid arthritis patients. Our study extends these findings to the context of radiation-induced intestinal inflammation.

The transport and bioavailability of Et to the wounded jejunum tissue may be improved by encapsulating it in nanostructured lipid carriers (Et-NLC).

Et-NLC can hinder the activation of p53 and cell death brought on by radiation in the epithelial cells of the jejunum. It may decrease NF-κB signaling, suppressing the production of inflammatory cytokines such as IL-6 and TNF-α generated by radiation. Radiation can kill cells in the epithelial cells of the jejunum, but Et-NLC can prevent p53 from activating and stopping cell death. It may decrease NF-κB signaling, suppressing the production of inflammatory cytokines such as IL-6 and TNF-α generated by radiation. These findings align with other research showing the potential of COX-2 inhibitors to reduce radiation-induced cell death and tissue damage^39^. Furthermore, the different levels of miR34a found in our study give us important information about the control mechanisms that govern how cells react to radiation and the possible protective effects of Et-NLC-based treatment. MiR34a, known for its role in apoptosis, proliferation, and stress response, exhibited significant alterations in expression across the experimental groups^42^.

The present study investigated the potential of etoricoxib-loaded nanostructured lipid carriers (Et-NLC) to reduce radiation-induced injury in rat jejunum Although the use of Et-NLC is an alternative, studies previously investigated the radioprotective effect of etoricoxib-only.

Consistent with our findings, studies have shown that etoricoxib can attenuate oxidative stress and radiation-induced inflammation^43^. Our results suggest that the Et-NLC formulation was able to further enhance these protective effects compared with etoricoxib alone, possibly due to better pharmacokinetics and targeted delivery of the drug.

Moreover, the observed changes in miR34a expression by Et-NLC treatment are consistent with the known regulatory roles of this microRNA in cellular stress responses. This indicates that Et-NLC systems may be greatly influenced by molecular mechanisms’ suitability.

Compared to previous studies on the use of etoricoxib for radioprotection, the present study provides new insights into the potential of nanocarrier-based delivery in clinical efficacy further studies are needed on these methods and their long-term benefits are well established.

However, when radiation was introduced, a remarkable upregulation of miR34a was observed in the radiation-only group (R). This elevation in miR34a expression may indicate the cellular response to radiation-induced stress and DNA damage^42^. Majidinia and Yousefi^44^ suggested that the high levels of miR34a are aligned with its role as a regulator of how cells respond to DNA damage and how they die. These results emphasize miR34a’s importance as a possible biomarker for radiation-induced damage. In contrast, the group treated with Et-NLC following radiation displayed a reduction in miR34a expression compared to the radiation-only group. This drop in miR34a levels might point to a potential protective effect of Et-NLC against radiation-induced stress. NLC has previously demonstrated the ability to enhance drug delivery and modulate cellular responses^24^. The specific mechanisms through which Et-NLC may influence miR34a expression warrant further exploration. Because of these results, more research is needed on the molecular processes that control miR34a regulation and how they protect and damage tissues exposed to radiation. Additional research is required to fully understand miR34a’s functions and promise as a diagnostic or therapeutic target in radiation cancer.

## Conclusion

The study’s findings suggest that using Et-NLC, which are nanostructured lipid carriers loaded with etoricoxib, could lessen the damage that radiation does to the rat jejunum. The histopathological analysis demonstrated that the jejunum suffered extensive damage as a result of radiation exposure. However, the application of Et-NLC following radiation treatment resulted in a notable enhancement in tissue integrity. In addition, the study investigated how radiation and Et-based treatments affect the regulation of the p53-miR34a axis. MiR34a is a crucial regulator of cellular stress responses and apoptosis, and its expression changes showed dynamic features. In general, Et-NLC shows promise as a radiation mitigator, protecting the jejunal tissue from radiation damage. Additional investigation is required to clarify specific molecular mechanisms and their enduring consequences.

## Materials and methods

### Resources

Etoricoxib (Et) was sourced from a generic drugstore (Merck & Co., Inc.). Analytical grade stearic acid, polysorbate 80, and oleic acid were procured from Sigma-Aldrich Co. All other compounds were of analytical grade.

### Dispersion and characterization of Et-NLC and their preparation

The Et-NLC dispersion was prepared using the low-temperature melt emulsification solidification method outlined by Sachan et al.^45^. The methanol-dissolved Et was combined with an acetone solution containing stearic and oleic acids. This mixture was added to a polysorbate 80 solution and sonicated for 15 min. Subsequently, the ingredients were combined using a mixer set at 3000 rpm and heated to 70 °C for 45 min. After cooling in an ice-water bath, the mixture was stirred at 3000 rpm for four hours. The NLC dispersions were then lyophilized overnight at − 50–55 °C and 0.05 mmHg using a Mitsubishi Electric Got 2000 zi33us unit to prepare them for further investigation. Following our previous research methodology^43^, the Et-NLC dispersions were characterized using zeta potential, particle size, polydispersity index distribution, XRD analysis, FTIR, and scanning electron microscopy (SEM). In our previous publication^43^, we extensively investigated the properties of the materials used in the current research, focusing on characterizing their physicochemical attributes, stability, and drug release profiles.

### Experimental animal groups

All studies were carried out in compliance with the ARRIVE criteria for reporting animal research. Thirty-six female Wistar albino rats weighing 180–200 g and 6–8 weeks’ old were obtained from VACSERA’s animal unit in Egypt. All rats were handled and treated according to Ethical Research Laboratory Animal Care Guidelines. The rats were divided into six groups using random allocation, comprising six (*n* = 6). **Group 1** (the control) received oral administration of normal saline for 14 days. **Group 2** (Et) received Et orally at a 10 mg/kg body weight dosage for 14 days using our established protocol^43^. **Group 3** (Et-NLC) was administered oral Et-NLC at 10 mg/kg body weight for 14 days^43^. **Group 4** (R) was exposed to a single dose of whole-body gamma radiation (6 Gy) and administered normal saline (0.5 ml/100 g body weight (BW) orally once daily for 14 days. **Group 5** (Et-R) received the same radiation dose of 6 Gy as Group 4, and then they received Et treatment similar to Group 2. Finally, **Group 6** (Et-NLC-R) was subjected to gamma radiation using the same procedure as Group 4, and then Et-NLC was administered, similar to Group 3.

### Gamma radiation

The gamma-caesium-137 radiation was conducted using a cell-40 irradiation unit (Atomic Energy of Canada Limited; Sheridan Science and Technology Park, Mississauga, Ontario, Canada) at the National Centre for Radiation Research and Technology (NCRRT) in Cairo, Egypt. The rats were exposed to a radiation dose of 0.33 Gy/min from a cesium-137 source located 1 m away. The radiation was applied bilaterally, with equal doses delivered from superior and inferior directions. The dosage was quantified using a radiophotoluminescence glass dosimeter (GD-302 M; Chiyoda Technol Co., Tokyo, Japan). The rats were exposed to a single dosage of 6 Gy inside custom-made acrylic containers. This radiation exposure was intended to generate oxidative stress, activate the inflammatory response^46^, and injure the jejunum^47^.

### Preparation of samples

The rats were injected with ethyl carbamate (urethane) intraperitoneally (IP) for animal anesthesia and then euthanized humanely by decapitation to collect samples precisely twenty-four hours after the last administration of either Et or Et-NLC. Blood samples were collected without using anticoagulants and then spun at a speed of 3000 rpm for five minutes to extract serum. The jejunum tissue of the rats in each group (10% wt/v) was immediately homogenized using an IKA homogenizer from Germany. The tissue was flushed with ice-cold PBS (0.01 M, pH = 7.4). The homogenates were centrifuged at a speed of 5000 rpm for 15 min at 4 °C using a Beckman Coulter Allegra 64R fixed-angle rotor (PN 392050). When the mixture was spun very quickly, the supernatant was used to measure the amounts of glutathione S-transferase (GST) activity, total protein (TP), malondialdehyde (MDA), superoxide dismutase (SOD), and nitric oxide (NO).

### Biochemical parameters in the serum

#### Liver and kidney function

We used commercially available test kits from Vitro Scient Co. in Cairo, Egypt, to determine serum alanine aminotransferase (ALT) enzyme levels, aspartate aminotransferase (AST) enzyme, and creatinine. The serum urea level was quantified using colorimetric analysis using commercially available test kits obtained from Vitro Scient Co. in Cairo, Egypt.

### The biochemical parameters in the jejunum homogenate

#### Evaluation of oxidative stress biomarkers

The spectrophotometric kits (Bio-diagnostic, Cairo, Egypt) were used to measure the activities of SOD (Cat. No. SD2521), GST (Cat. No. GT2519), and MDA (Cat. No. MD2529) in the intestinal tissue, following the manufacturer’s recommendations. Rat ELISA NO kit (MBS2604161) was purchased from MyBioSource (San Diego, CA, USA) and performed according to the manufacturer’s instructions.

#### DNA fragmentation

The genomic DNA from the jejunum samples was extracted and purified using the GeneJet Genomic DNA Purification Kit (Thermo Scientific, #K0721), following the instructions provided by the manufacturer. The length of DNA fragments was estimated using a two-kilobase pair (kbp) DNA ladder (PeqGold 2Kb, Peqlab, GMH). The genomic fragments were placed on a 1.5% (w/v) Agarose gel, treated with ethidium bromide, separated using electrophoresis (75 V, 150 mA), and seen on a UV plate. The data analysis was performed using a gel-based recording device, Geldoc-it, manufactured by UVP in England. The TotalLab analysis program (Ver.1.0.1) from http://www.totallab.com was used for this purpose.

#### miR34a rat microRNA expression plasmid (MI0000877)

##### RNA extraction

The jejunum tissue lysate was subjected to RNA extraction using the Direct-zol RNA Miniprep Plus kit (Cat# R2072, ZYMO RESEARCH CORP., USA). Subsequently, the amount and quality of the isolated RNA were evaluated using a Beckman dual spectrophotometer (USA). The Direct-zolTM RNA Miniprep Plus offers an efficient technique for isolating high-quality RNA directly from samples in TRIzol^®^. This method allows for the purification of up to 100 µg of RNA per preparation. It can effectively isolate total RNA, including small RNAs ranging from 17 to 200 nucleotides, from tissue and samples stored in DNA/RNA Shield™. Ethanol was added to a TRI Reagent^®^ sample and immediately attached to the Zymo-SpinTM Column. The sample was rinsed, and RNA was then eluted. The RNA is of excellent quality and is prepared for next-generation sequencing, RT-qPCR, transcription profiling, hybridization, and other applications.

##### The real-time polymerase chain reaction (RT-PCR)

The RT-PCR was performed using the SuperScript IV One-Step RT-PCR kit (Cat# 12594100, Thermo Fisher Scientific, Waltham, MA, USA). This kit was used for the reverse transcription of the extracted RNA, followed by PCR in a single step. The InvitrogenTM SuperScriptTM IV One-Step RT-PCR System is specifically designed for susceptible detection and analysis of RNA by reverse transcription polymerase chain reaction (RT-PCR). cDNA synthesis and PCR amplification were performed in a single reaction tube using gene-specific primers (Table 2). SuperScriptTM IV RT Mix was used for cDNA synthesis. The reverse transcription (RT) reaction was carried out under optimized conditions using M-MULV enzyme. The SYBR Green dye fluoresces upon binding to double-stranded DNA.

**Table 2:** Primers used for gene expression detection in rats.

Gene	Primer sequence
miR34a	FW: 5′-CCGGCTGTGAGTAA-3′ RV: 5′-GTGCTGCACGTTGT-3′
GAPDH	FW: 5′-TGGATTTGGACGCATTGGTC-3′ RV: 5′-TTTGCACTGGTACGTGTTGAT-3′

##### Relative quantification (RQ)

The quantification of RQ, also known as relative expression, is being calculated. Following the completion of the RT-PCR run, the results were quantified using the cycle threshold (Ct) method. The PCR data sheet contains the Ct values of the target gene compared to the reference gene (GAPDH). Utilizing a control sample is crucial for quantifying the gene expression of a particular gene. The housekeeping gene is used to determine each target gene’s relative quantification (RQ), which is done by computing the delta-delta Ct (ΔΔCt). The RQ of each gene was determined using the formula 2^−∆∆Ct^.

#### Western blotting technique

We used jejunum tissue homogenates for Western blotting analysis to check COX-2 [(29): sc-19999], IL-6 [(10E5): sc-57315], IL-10 [(E-10): sc-8438], TNF-α [(52B83): sc-52746], and P53 [(DO-1): sc-126] levels. The ReadyPrepTM protein extraction kit from Bio-Rad Inc. (Catalog #163-2086) was used to extract total protein from homogenized intestinal tissues of various groups. The Bradford Protein Assay Kit from Bio Basic Inc. (Markham Ontario L3R 8T4 Canada) was used for quantitative protein analysis. Twenty micrograms of protein were mixed with twice as much Laemmli sample buffer to ensure they were denatured before being put on the polyacrylamide gel for electrophoresis. Bio-Rad Laboratories Inc. supplied polyacrylamide gels using the TGX Stain-FreeTM FastCastTM Acrylamide Kit (SDS-PAGE) (Cat # 161-0181). The electrophoresis was conducted for 7 min at a voltage of 25 V. Next, a solution with TBST buffer, 3% bovine serum albumin (BSA), and tris-buffered saline with Tween 20 was added to the blotting membrane to stop non-specific binding.

After electrophoresis, proteins were transferred to a PVDF membrane using a transfer buffer (Towbin buffer) at 100 V for 1 h. The membrane was then blocked in TBST buffer (tris-buffered saline with Tween 20) containing 3% bovine serum albumin (BSA) for 1 h at room temperature. They were blotted with primary antibodies directed against the COX-2 (dilution 1:1000), IL-6 (dilution 1:1000), IL-10 (dilution 1:1000) TNF-α (dilution 1:500), P53 (dilution 1:1000). Incubation was performed overnight at 4 °C. The membrane was washed with TBST and then incubated with HRP-conjugated secondary antibody (Goat anti-rabbit IgG-HRP-1 mg Goat mab, Novus Biologicals) for 1 h at room temperature. Development of chemiluminescent signal after washing Clarity TM Western ECL substrate (Bio-Rad catalog no. 0-5060) was detected using a camera equipped with a CCD camera. Image analysis software was used to read the band intensity of the target proteins against control sample beta actin (housekeeping protein) by protein normalization on the ChemiDoc MP imager.

### Pathological analysis

Samples of jejunum tissue were preserved in a 10% formal salt solution, then fractionated, cleaned, and dehydrated in a graded series of alcohols: (a) Wash in 70% alcohol for 1 h. (b) Wash in 95% alcohol for 1 h (two times). (c) Wash in 100% alcohol for 1 h (two times). The dehydrated samples were treated with xylene to remove residual alcohol and then embedded in paraffin blocks for preservation. Sections of 4–6 μm thickness were cut from the paraffin sections. Under light microscopy, these sections were stained with hematoxylin and eosin (H&E) for histological examination^48^. The severity of intestinal lesions was assessed on a semiquantitative scale using the following criteria: (1) degeneration of the surface and crypt epithelium; (2) degeneration of the villus structure, and (3) inflammatory cell infiltration. Each criterion was assigned a score ranging from 0 (no lesions) to 3 (severe lesions), with 1 and 2 representing mild and moderate lesions, respectively. The researchers obtained five pathological sections from each rat and evaluated the microscopic score of the ileum by summing the values assigned to each criterion. They investigated at least five tiny locations for each specimen^49^.

To minimize potential bias, all histopathological evaluations were conducted by experienced pathologists who were blinded to the treatment groups.

### Statistical evaluation

Result findings are shown as mean values with standard error for six animals. A parametric test was utilized to establish if the hypothesis was correct. The normality of data distribution was assessed using the Shapiro-Wilk test. The data were found to have a normal distribution, allowing for the use of parametric tests. A parametric test was utilized to establish if the hypothesis was correct. Several assessments were statistically analyzed using one-way ANOVA testing and Tukey’s HSD multiple comparisons as a post-hoc test to discover significant differences between groups. *p* < 0.05 was chosen as the statistically significant level. For all statistical analyses, the SPSS statistical version 23 (SPSS^®^ Inc., USA) was utilized.

### Approval of the study’s ethical considerations

The animal treatment protocol was approved by the Animal Care Committee of the National Center for Radiation Research and Technology (NCRRT), Cairo, Egypt (REC-NCRRT-13 A/22). This research was done in compliance with the ARRIVE guidelines and regulations (https://arriveguidelines.org).

## Electronic supplementary material

Below is the link to the electronic supplementary material.

Supplementary Material 1

## Data Availability

The data used or analysed during the current study are available from the corresponding author on reasonable request.
