# Supplementary material for: Mitigation of radiation-induced jejunum injuries in rats through modulation of the p53-miR34a axis using etoricoxib-loaded nanostructured lipid carriers
Source: Sci Rep. 2024 Oct 10;14:23728. doi: 10.1038/s41598-024-73469-7 (PMC11467169; doi:10.1038/s41598-024-73469-7)

Mitigation of Radiation-Induced Jejunum Injuries in Rats through Modulation of the p53-miR34a Axis Using Etoricoxib-Loaded Nanostructured Lipid Carriers

Supplementary Data

Supplementary fig. S1 (relative to fig. 2)

Loading order: Control, R, Et, Et-NLC, Et-NLC+R, Et+R

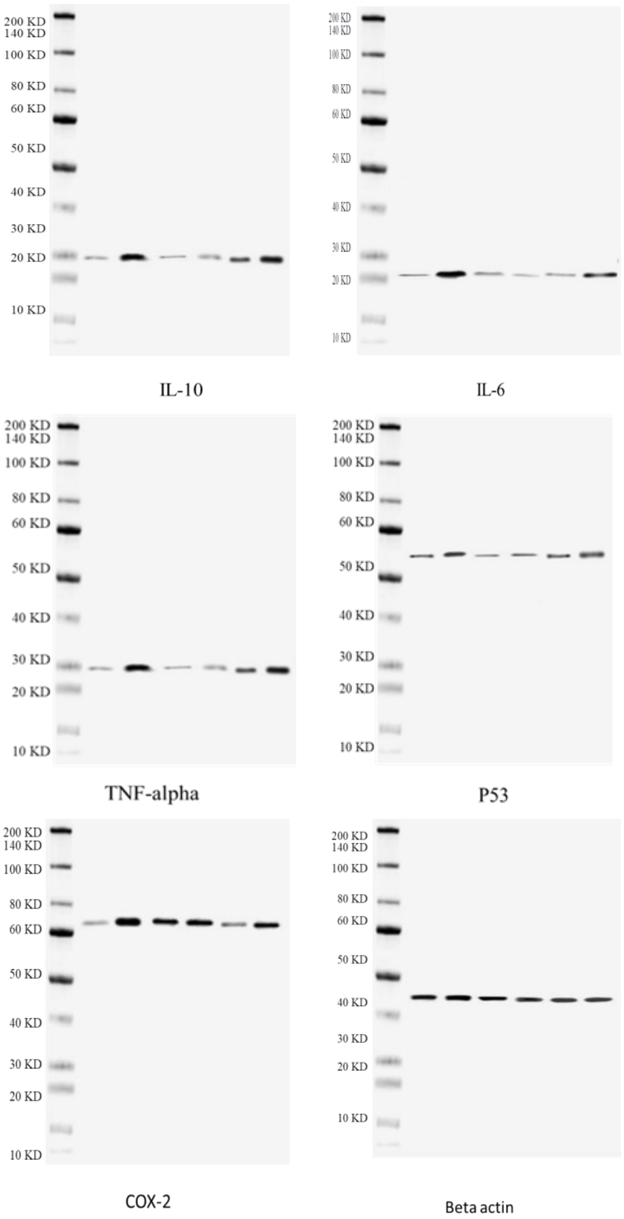

Supplementary fig. S2 (relative to fig. 3)

Fig. S2a: dendogram of DNA ladder.

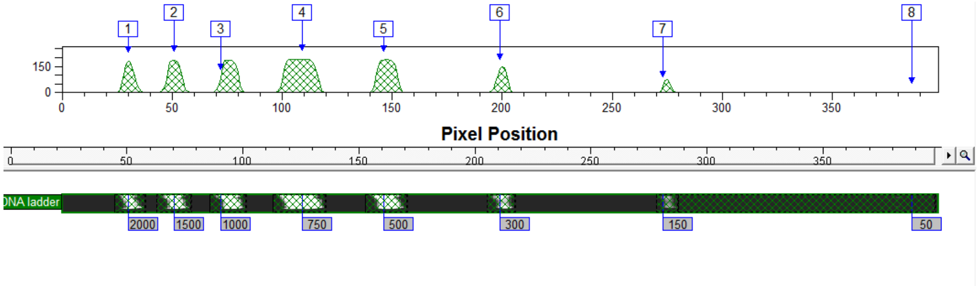

Fig. S2b: dendogram of Control (C)

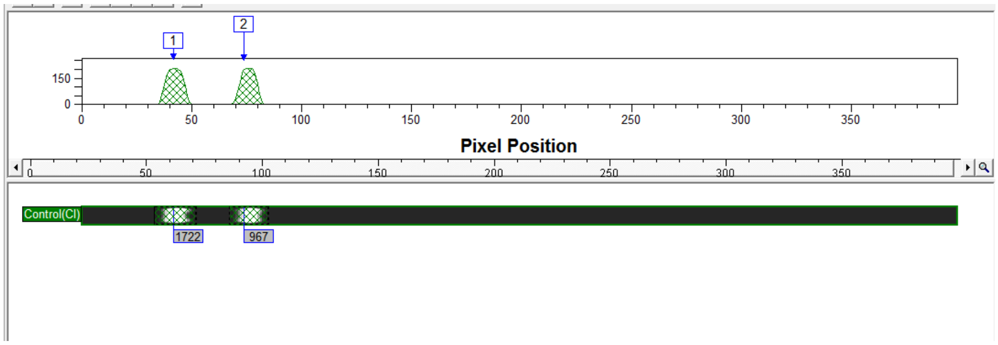

Fig. S2c: dendogram of Radiation (R)

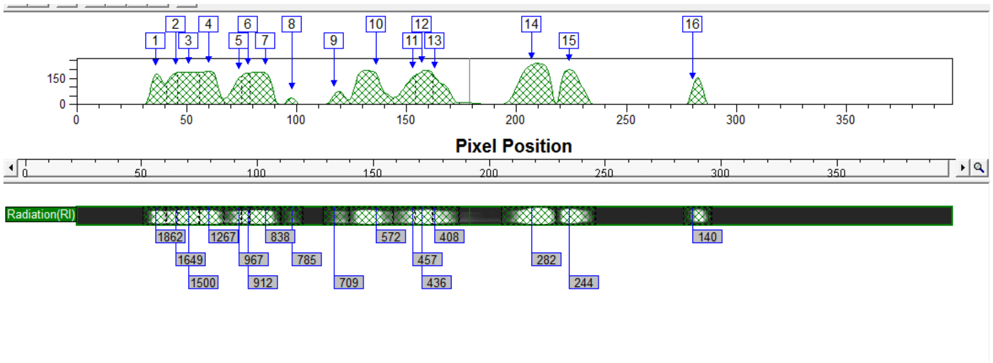

Fig. S2d: dendogram of Et

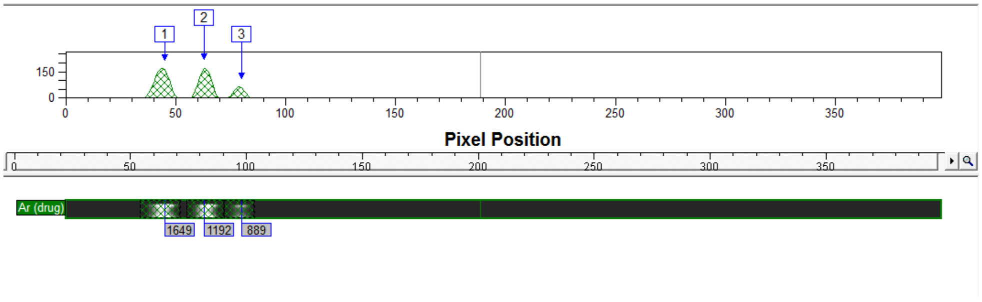

Fig. S2e: dendogram of Et-NLC

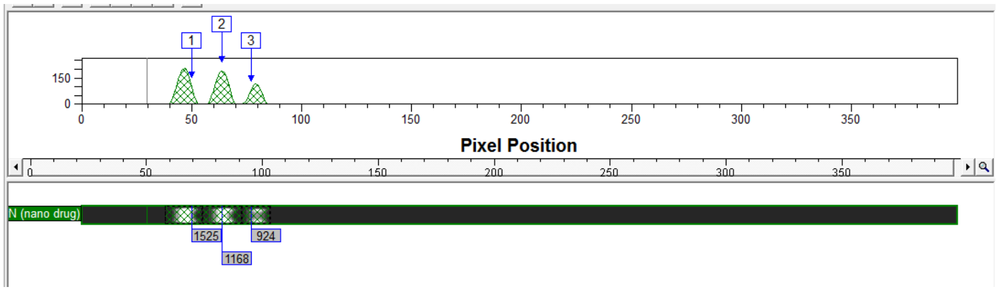

Fig. S2f: dendogram of Et-NLC+R

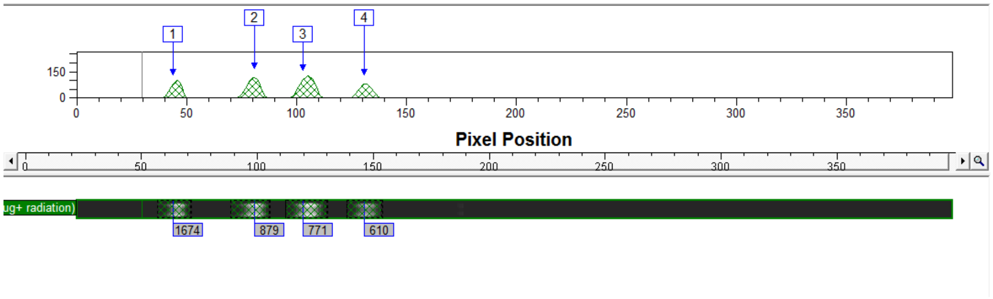

Fig. S2g: dendogram of Et+R

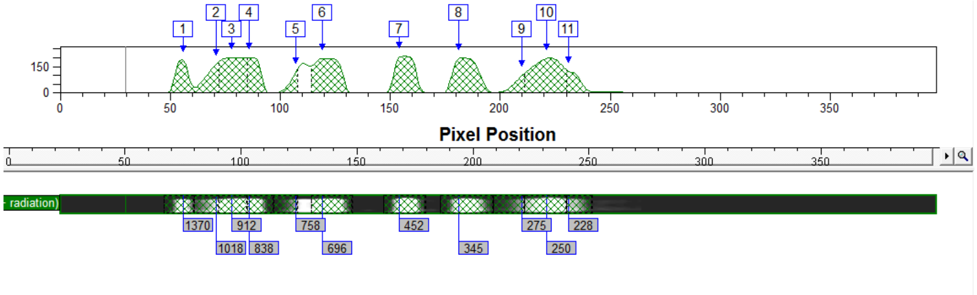

Supplement: Supplementary file 1 — Supplementary Material 1 [file 41598_2024_73469_MOESM1_ESM.pdf]
